# The Book World of Medicine and Science

**Published:** 1903-11-28

**Authors:** 


### THE BOOK WORLD OF MEDICINE AND SCIENCE.

Text-Book of Operative Surgery. By Th. Kocher, M D., Bern. Pp. 440. Illustrations, 255. Price 20s. net. (London: Adam and Charles Black. 1903.)

This is a translation of the fourth (the latest) German edition, and is the second English edition. At the outset the most sincere thanks must be tendered to Mr. Harold J. Stiles for his energy in presenting the work in English, especially as this edition has nearly twice as many pages as the first. So much, moreover, has been rewritten by the author, that the work has become almost a new book. Professor Kocher's name and work are rightly held in the highest esteem both abroad and in Great Britain, and the placing again of his treatise in the hands of English surgeons and students in their mother tongue cannot fail to be productive alike of interest and enlightenment. There is something in the masterly and orderly manner in which the writer deals with his subject which is fascinating to the scientific mind, and the brilliancy of the translation has enabled the English reader to lose none of the inspiration of the original. A word concerning the illustrations should be given here. They number 255, and are excellent. In certain, as for instance many indicating the procedure in amputations and excisions, colouring of muscles has been introduced, and all arteries and veins have been appropriately tinged in sections dealing with vessels. There are, however, one or two parts in which it is to be regretted that illustrations are absent, none appearing for example, in the otherwise excellent account of the operations necessary for carcinoma of the rectum. Perhaps the most graphic and original are those indicating the steps for excision of a goitre, in which operation the author is an untouched master. It is somewhat surprising and disappointing that the whole of the operative manipulations for enlargement of the prostate is dealt with in less than the compass of a single page. There is a lucid account of the author's own method of radical operation upon both inguinal and femoral herniæ, and with true consistence, the outcome of experience of no mean order, he upholds the superiority of it to those operations which involve the opening of the inguinal canal and the ligature of the neck of the sac flush with the parietal peritoneum. Excisions of the individual bones of the foot and the various operations for talipes are extremely well described and illustrated, but there is wanting any account of the modern methods of tendon transplantation. Excision of the patella is given a place, and the author states that the results are very satisfactory, a completely movable joint being obtained. Excision of the innominate bone is one of the operations which we believe was first performed by Professor Kocher, and he also alludes to a case of total resection of the sacrum for suppurative carionecrosis. In the matter of amputations the writer advocates an internal rather than a true heel-flap in a modified Syme, because there is so much less space left between the divided bones and the soft tissue of the flap; and in amputation through the leg suggests an ingenious osteo-plastic manœuvre. The book terminates with an appendix on the freeing of the duodenum and gastroduodenostomy, which well repays perusal. The work is well printed, and has an excellent index, and is deservedly worthy of recommendation.

Animal Physiology Diagrams, with an Object Lesson Handbook. By Walker Overend, M.D., B.Sc. (London: Thomas Nelson and Sons. Price 20s.)

This publication consists of ten large sheets devoted respectively to illustrations of the skeleton, muscles, teeth, organs of digestion, circulation of the blood, organs of respiration, kidneys and skin, nervous system, eye, and ear. On each sheet are a few lines of descriptive letterpress. In addition there is provided for the use of lecturers a small handbook which contains hints on such points as may be usefully dealt with apropos of each sheet of illustrations. We have before now had occasion to insist on the value of good diagrams for teaching purposes, and those now before us will no doubt prove to be of the greatest service to all who give lectures on anatomy and physiology to classes of students whose previous knowledge of these subjects has not passed far beyond an elementary stage. The diagrams are of an ample size for lecture purposes, and they are for the most part as accurate as possible without at the same time being made too complicated by unnecessary detail There are one or two small matters which it might be well to alter in a future edition; for example, in the first page illustrating the component parts of the skeleton, the spinal cord is depicted as though it reached to the sacrum, whereas in the adult it usually does not extend further than the second lumbar vertebra. Again in the sheet portraying the human organs of digestion the term "ileum" is used as though it could be applied to the whole length of the small intestine. It is, perhaps, to be regretted that space has not been found for dealing with the lymphatic system. However, these are very trifling defects, and do not materially affect the high general standard of excellence of the illustrations or their usefulness for teaching purposes.

First Aid. By R. J. Collie, M.D., and C. F. Wightman, F.R.C.S. (London: George Gill and Sons. Pp. 125. Price 6d.)

This is an excellent little manual and should prove of much use to those who wish to obtain a sound knowledge of the principles of first aid, and especially to those who are attending a course of lectures on the subject. The arrangement of the book is into chapters, which correspond to the lectures set forth in the syllabus of the London School Board. A valuable feature is the copious supply of illustrations from photographs which very greatly facilitate a proper understanding of the text. The book is of handy size, has been carefully prepared, and the style is particularly simple and lucid, a matter of chief importance in a work of this kind.

Practical Advertising, 1903–4. (Published by Mather and Crowther, Limited, London).

The 1904 edition of this useful guide contains, as before, a preface of articles on subjects of interest to advertisers, followed by very complete lists of newspapers published throughout the Empire. We also notice the addition of a list of Bill Posters in the United Kingdom to the present issue. The book is well produced and should prove exceedingly useful to those for whom it is designed.

